# An Introduction to Dermatology

**Published:** 1899-11

**Authors:** 


AN INTRODUCTION TO DERMATOLOGY. By NORMAN WALKER, M. D., Fellow of the Royal College of Physicians of Edinburgh; Assistant Physician for Diseases of the Skin to the Royal Edinburgh Infirmary. With a frontispiece, twenty-nine plates and thirty-four illustrations in the text. Octavo volume, 250 pages. Price, $3.00. New York: William Wood & Co.

A book of this kind may find readers among physicians, but should not among students. It conveys much of the latest information concerning the subject of which it treats, even upon disputed points, a fact which might lead the mere student to dangerous conclusions. It was the publisher's idea to prepare the best and cheapest rudimentary book in the English language. Of course no publication can readily usurp the place occupied by Jackson's masterly hand-book upon Diseases of the skin, or successfully compete with Hardway's Manual. Had this author given the essentials of all diseases, refraining from the expression of mere personal experience, his book would have been stronger, more reliable and more useful. Furthermore, his quotations from Unna are quite too frequent, betraying more partiality than discretion. In short, the work is not for the student, but for such physicians as are especially interested in the subject treated. The whole manner and matter of the book is far too advanced to justify its claims to be considered as a treatise upon introductory dermatology.

A striking feature of the work consists in the limited number of American authors quoted. This is unjust to the very many who have done so much in establishing the well formulated principles of dermatology. The only person who has been freely quoted outside of Great Britain, is Unna—perhaps the author thinks "his friend" has been quoted sufficiently to atone for his neglect of scores of others equally great, if not actually greater.

It is curious to notice that no mention is made of blastomycetic dermatitis, porokeratosis, epidermolysis, bullosa, hydrocystoma and various other important skin affections. The book is large enough to have included the rarer as well as the really common dermatological subjects.

Although the chapter upon eczema must be regarded as the best in the book, since eczema represents a large percentage of all diseases of the skin, too little is said about the elements of the affection, while the actual definition, in spite of the author's protest, is at once sarcastic and inadequate. Parasitic diseases receive considerable consideration. Pediculosis vestimentorium is placed among the hemorrhages; while pediculosis capitis is put down among the infectious inflammations; places pityriasis versicolor and erythrasma under the heading of sapropytes; while scabies, favus and ringworm are ranked with pediculosis capitis.

The article on acne, in giving the cause of the affection, lays too much stress upon the microörganism as the cause, while the more rational reasons are feebly given; and to consider ichthyosis as an infectious disease is without warrant. Neither will it do to characterise psoriasis and seborrheal dermatitis as virtually the same disease.

The article on ringworm is nearest complete, and the treatment suggested is fairly good, but the classification tends to mislead, as the author in his servile imitation of Unna denies himself the benefit of suggestions from others quite as capable. The book is sadly unequal—some diseases are fairly and fully treated while others have received only the most meager consideration. For instance, only four pages have been devoted to syphilis; it would have been better had nothing been said upon the subject. Symptomatology has received inadequate consideration throughout the entire book; histology has received more than can be found in any other book of its size; diagnosis is fairly well presented. Treatment is the strongest part of the book and has been carefully considered. The volume is written in clear, direct style. The author belongs to the new school—perhaps too new to be really valuable to the learner.

The importance of pictures he clearly recognises, in a work on dermatology; most of these in the book represent cases of his own. A number of the half-tones are good and are well chosen. The flesh tints of the chromo-lithographs are too decided, although they do not fail to aid in illustrating their subjects.

William Wood & Co. have produced this volume in their characteristic style, which leaves nothing to be desired. G. W. W.

